# Identification of the Prognosis-Related lncRNAs and Genes in Gastric Cancer

**DOI:** 10.3389/fgene.2020.00027

**Published:** 2020-02-11

**Authors:** Xiaohui Su, Jianjun Zhang, Wei Yang, Yanqing Liu, Yang Liu, Zexing Shan, Wentao Wang

**Affiliations:** Department of Gastric Surgery, Cancer Hospital of China Medical University, Liaoning, China

**Keywords:** gastric cancer, long noncoding RNA, prognostic, TCGA, model

## Abstract

Gastric cancer is a common malignant tumor with high occurrence and recurrence and is the leading cause of death worldwide. However, the prognostic value of protein-coding and non-coding RNAs in stage III gastric cancer has not been systematically analyzed. In this study, using TCGA data, we identified 585 long noncoding RNAs (lncRNAs) and 927 protein-coding genes (PCGs) correlated with the overall survival rate of gastric cancer. Functional enrichment analysis revealed that the prognostic genes positively correlated with death rates were enriched in pathways, including gap junction, focal adhesion, cell adhesion molecules (CAMs), and neuroactive ligand-receptor interaction, that are involved in the tumor microenvironment and cell-cell communications, suggesting that their dysregulation may promote the tumor progression. To evaluate the performance of the prognostic genes in risk prediction, we built three multivariable Cox models based on prognostic genes selected from the prognostic PCGs and lncRNAs. The performance of the three models based on features from only PCGs or lncRNAs or from all prognostic genes were systematically compared, which revealed that the features selected from all the prognostic genes showed higher performance than the features selected only from lncRNAs or PCGs. Furthermore, the multivariable Cox regression analysis revealed that the stratification with the highest performance was an independent prognostic factor in stage III gastric cancer. In addition, we explored the underlying mechanism of the prognostic lncRNAs in the Cox model by predicting the lncRNA and protein interaction. Specifically, *CTD-2218G20.2* was predicted to interact with *PSG4, PSG5,* and *PSG7*, which could also interact with cancer-related proteins, including *KISS1, TIMP2, MMP11, IGFBP1, EGFR,* and *CDKN1C*, suggesting that CTD-2218G20.2 might participate in the cancer progression *via* these cancer-related proteins. In summary, the systematic analysis of the prognostic lncRNAs and PCGs was of great importance to the understanding of the progression of stage III gastric cancer.

## Introduction

Gastric cancer is one of the most commonly diagnosed cancers worldwide, with an incidence of 1,031,700 new cases in 2018 and poor survival rates, causing approximately 787,200 deaths that year (Bray et al., 2018). Incidence rates of gastric cancer exhibit significant differences among regions, as its rates in Eastern Asia are markedly higher than those in Northern America and Northern Europe, and about 70% of gastric cancer is reported in developing countries with a higher mortality ratio, reflecting the importance of modern surgical and medical technology in gastric cancer treatment (Guggenheim and Shah, 2013). Environmental risk factors for gastric cancer include Helicobacter pylori infection, tobacco and alcohol use, and dietary salt intake (Zhang and Zhang, 2017), while genetic studies have revealed several key genetic factors in gastric cancer, including chromosomal instability, changes in microRNA profile, and somatic gene mutations (McLean and El-Omar, 2014).

According to the TNM system, most of GC patients are suffering from stage III or stage IV disease (Washington, 2010; Coburn et al., 2018). Surgery may seem to be the only approach to ensure long-time survival; however, for patients who have undergone surgical resection, the recurrence-free survival time remains poor, with a median length shorter than two years (Spolverato et al., 2014; Chan et al., 2016). Although adjuvant radiotherapy and chemotherapy are utilized to reduce its recurrence after surgery, the five-year survival rate for all stages is still unsatisfying, as it merely becomes 65% for patients with stage I disease, and the situation is much worse for patients with more advanced stages (Spolverato et al., 2014).

The discovery of biomarkers will greatly help deliver personalized treatment, with the goal of reducing gastric cancer recurrence and mortality rates. Currently, most studies investigating biomarkers in gastric cancer focus on protein-coding genes (PCGs), but noncoding RNAs are less addressed (Nagarajan et al., 2012). Though a growing number of long noncoding RNAs (lncRNAs), including *HOTAIR, MEG3, MALAT1, H19, GAPLINC,* and *GClnc1*, have been reported to be associated with gastric cancer tumorigenesis, the role of lncRNAs in human gastric cancer and their prognostic value are still inadequately explored (Kogo et al., 2011; Gu et al., 2015; Sun et al., 2016). Furthermore, the performance of the protein-coding genes and lncRNAs in risk prediction has not been systematically compared in gastric cancer. In the present study, we collected gene expression data for stage III gastric cancer and aimed to identify key prognostic lncRNAs in gastric cancer. Moreover, we built a Cox model based on features from both protein-coding genes and lncRNAs and compared the performance of Cox models based on features from prognostic lncRNAs, from pPCGs, and from all prognostic genes. The systematic analysis of the prognostic lncRNAs and PCGs is of great importance for the understanding of the progression of stage III gastric cancer.

## Materials and Methods

### TCGA Gene Expression Data Collection and Processing

The gene expression data of TCGA stomach adenocarcinoma (TCGA-STAD) and the associated clinical data were downloaded from the UCSC Xena database (Goldman et al., 2018) (https://xenabrowser.net/datapages/). Samples diagnosed with TNM stage III in TCGA-STAD were selected for the downstream data analysis. Each gene was discretized as of high and low expression status if its expression level was higher or lower than the median, respectively. The survival analysis was conducted based on the discretized expression status.

### Overrepresentation Enrichment Analysis (ORA)

To characterize the prognostic genes, we employed overrepresentation enrichment analysis (ORA), which was implemented by R package *clusterProfiler* with the *enrichKEGG* and *enricher* functions (Yu et al., 2012). The gene sets used for the enrichment analysis of the lncRNA interacting proteins were collected from MSigDB gene sets (Liberzon et al., 2011) (http://software.broadinstitute.org/gsea/index.jsp). The significant pathways were selected based on a threshold of 0.05 for adjusted *P*-value.

### Cox Proportional Hazards Regression Analysis

The two-sample comparisons of overall survival were performed by Cox proportional hazards regression analysis and the differences tested by log-rank test, implemented using the R package *survival* with the *coxph* function. The predicted risk score for the patients was calculated based on the expression status of the prognostic genes, implemented in R with the *predict.coxph* function. Particularly, the features (prognostic genes) were selected by the Maximum Minimum Parents and Children (MMPC) algorithm (Lagani et al., 2016) and implemented by the *MXM* package in R.

### lncRNA–Protein Interaction Analysis

To predict the potential lncRNA–protein interactions, we used the pre-trained LncADeep (Yang et al., 2018) model, a deep learning model, and utilized the sequences of differentially expressed lncRNAs and proteins to predict their interactions. In addition, we also conducted Pearson correlation analysis between the lncRNAs and proteins, with a threshold of 0.3 for Pearson correlation coefficients (PCC).

### Statistical Analysis

The statistical analyses were conducted in R programming software, version 3.6.0. The two-sample or multiple-sample comparisons were performed using the Wilcoxon rank-sum test or analysis of variance (ANOVA). *P*-value < 0.05 was considered statistically significant difference.

## Results

### Identification of Prognostic Genes by a Univariable Cox Model

**Figure 1:**
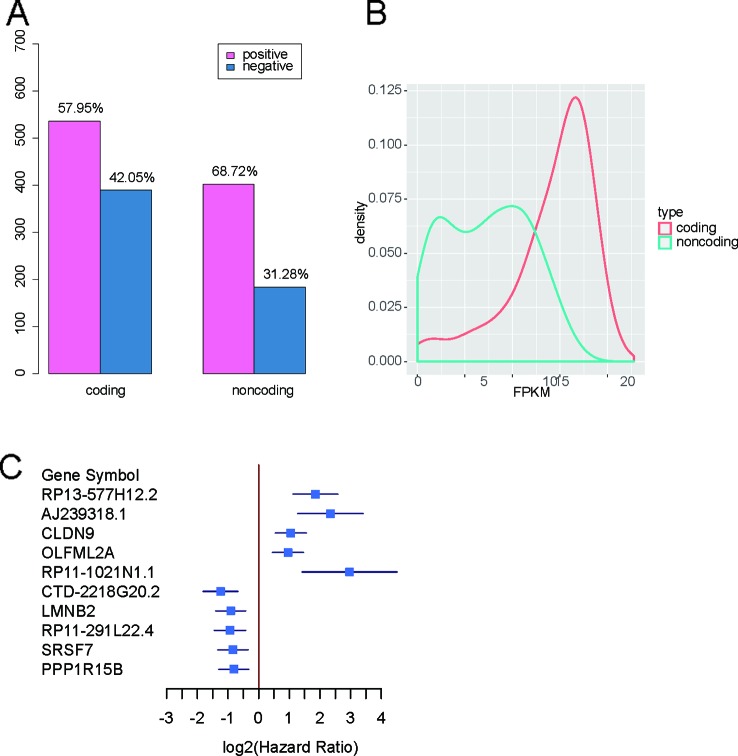
Overview of the prognostic protein-coding genes (PCGs) and long noncoding RNAs (lncRNAs). **(A)** Proportions of prognostic PCGs and lncRNAs positively and negatively correlated with death rates. **(B)** Distribution of expression levels for the PCGs and lncRNAs. **(C)** Forest plot for the top five genes positively and negatively correlated with death rates.

To identify the prognostic genes, including the long noncoding RNAs (lncRNAs) and protein-coding genes (PCGs), we collected 152 stage III gastric cancer samples from the TCGA gastric cancer cohort. The univariable survival analysis was then conducted on all the genes with stable expression (FPKM > 1 in 10% of samples). In total, we identified 585 lncRNAs and 927 PCGs correlated with overall gastric cancer survival (**Supplementary Table S1**). Notably, 57.95% of PCGs and 68.72% of lncRNAs positively correlated with death rates in the Cox models were identified (**Figure 1A**), suggesting that these genes might drive the cancer progression. The two proportions showed significant difference (two-sample proportion test, P < 0.05), which might be caused by the relatively lower expression of lncRNAs. Moreover, we also investigated the distribution of the prognostic gene expression levels. The prognostic PCGs had significantly higher expression than the prognostic lncRNAs (**Figure 1B**). As shown in **Figure 1C**, the top five genes positively and negatively correlated with death rates included *RP13-577H12.2, AJ239318.1, CLDN9, OLFML2A, RP11-1021N1.1, CTD-2218G20.2, LMNB2, RP11-291L22.4, SRSF7,* and *PPP1R15B*. Notably, *RP13-577H12.2, CTD-2218G20.2,* and *RP11-291L22.4* were prognostic lncRNAs.

### Functional Characterization of the Prognostic Genes

**Figure 2:**
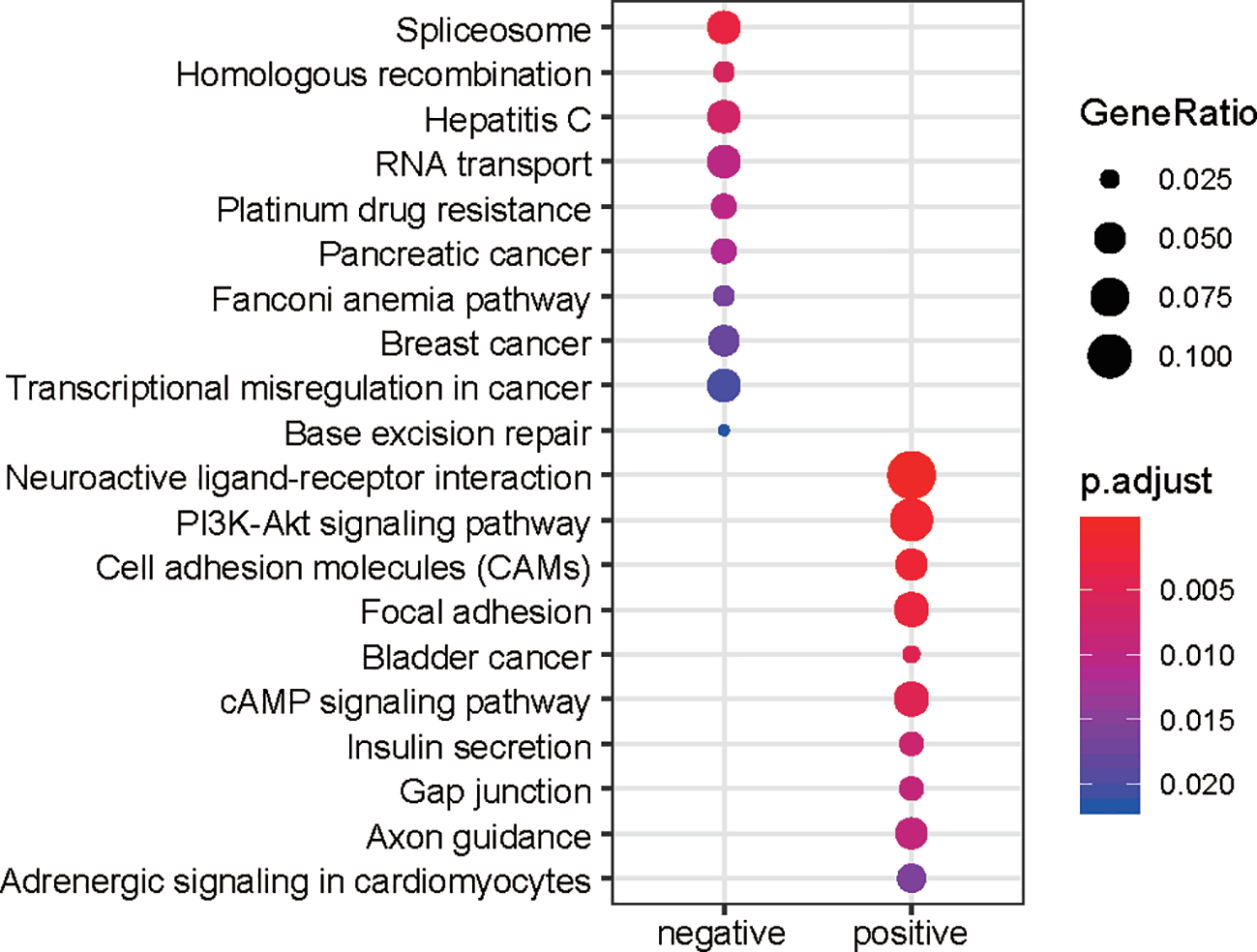
KEGG enrichment of the prognostic genes. The node size represents the ratio of the genes in the pathway. The colors represent the statistical significance of the pathways.

To characterize the functions of the prognostic genes, the prognostic genes positively or negatively correlated with death rates were subjected to KEGG enrichment analysis. The genes promoting the progression of gastric cancer were enriched in pathways such as adrenergic signaling in cardiomyocytes, axon guidance, gap junction, insulin secretion, the cAMP signaling pathway, bladder cancer, focal adhesion, cell adhesion molecules (CAMs), the PI3K-Akt signaling pathway, and neuroactive ligand-receptor interaction (**Figure 2**). In contrast, the genes with higher expression in samples with better prognosis were enriched in base excision repair, transcriptional misregulation in cancer, breast cancer, Fanconi anemia pathway, pancreatic cancer, platinum drug resistance, RNA transport, hepatitis C, homologous recombination, and spliceosome (**Figure 2**). It should be noted that the gap junction, focal adhesion, cell adhesion molecules (CAMs), and neuroactive ligand-receptor interaction pathways were involved in tumor microenvironments and cell-cell communications, suggesting that their dysregulation may promote the tumor progression.

### The Performance of the Prognostic lncRNAs and PCGs in Risk Prediction

**Figure 3:**
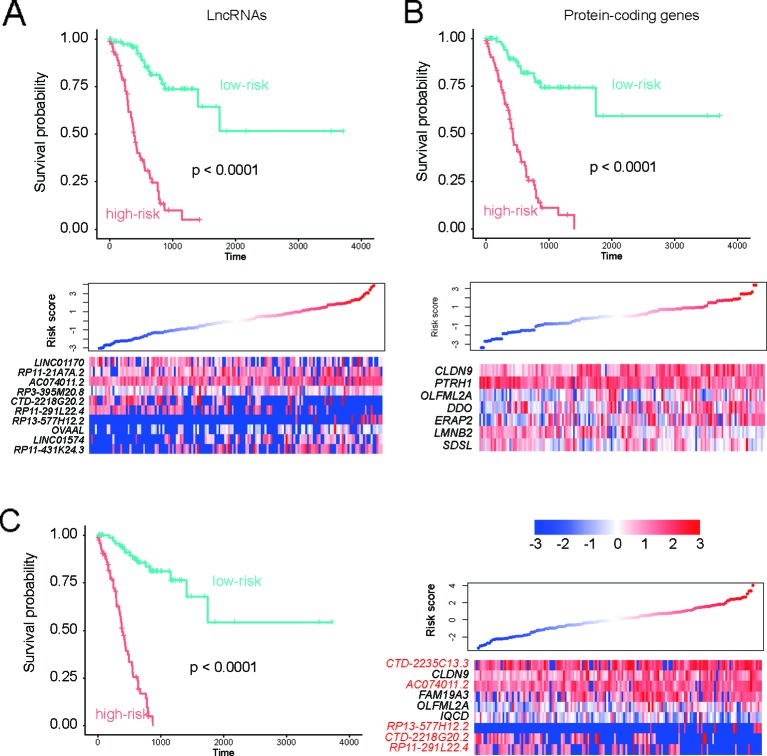
Performance of the three Cox models in risk prediction. Performance of three Cox models based on the features selected from only the prognostic lncRNAs, only the prognostic PCGs, and all the prognostic genes are displayed in **(A–C)**, respectively.

To evaluate the performance of the prognostic genes in risk prediction, we first selected features from the lncRNAs, PCGs, and all genes, respectively, with a significance level of 0.01 using the MMPC algorithm. Specifically, 10 lncRNAs and seven PCGs were selected for the construction of Cox models based on only lncRNAs or PCGs (**Figures 3A, B**). Additionally, another nine genes including five lncRNAs and four PCGs were selected to build the model under both lncRNAs and PCGs (**Figure 3C**). As shown in **Figure 3**, the risk groups stratified by the three Cox models showed significantly different overall survival (*P* < 0.0001), and the selected features were highly correlated with the risk. Furthermore, we also compared the performance of the three models based on the criteria of log-rank test, Wald test, and C-index (**Table 1**). Consistently, the features selected from all the prognostic genes showed higher performance than the features selected only from lncRNAs or PCGs (**Table 1**), suggesting that stratification by feature by integrating PCGs and lncRNAs was superior to using either of the two alone.

**Figure 4:**
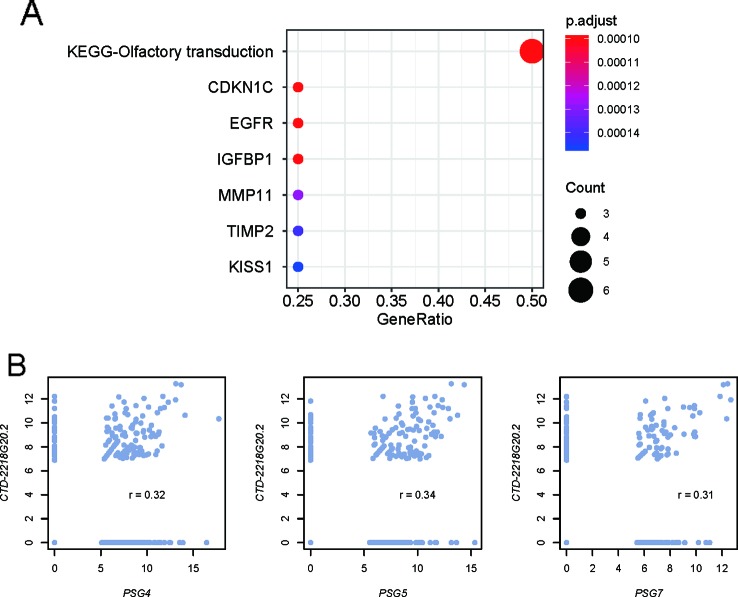
Predicted function of the prognostic lncRNA CTD-2218G20.2. **(A)** Gene sets enriched by the proteins predicted to interact with CTD-2218G20.2. **(B)** Correlation analysis between CTD-2218G20.2 and three pregnancy-specific glycoproteins. The x- and y-axes represent the expression levels (log2 (FPKM+1)) of the PSG genes and CTD-2218G20.2, respectively.

**Table 1:** Performance of three Cox models based on features selected from all genes, PCGs, and lncRNAs.

Features	logtest.pvalue	waldtest.pvalue	C-index	sd(C-index)
All genes	3.56E-20	1.48E-16	0.84	0.04
PCGs	2.73E-16	8.20E-14	0.80	0.04
LncRNAs	2.99E-18	8.59E-14	0.83	0.04

### The Stratification Based on the Features From All Genes Is an Independent Prognostic Factor in Stage III Gastric Cancer

As the prognostic model based on the features from all genes exhibited satisfying performance on all stage III gastric cancer patients, it was also necessary to investigate whether this stratification was a prognostic factor independent of clinical indicators such as age, gender, race, and histology grade. The multivariable Cox regression model was then constructed by group and these co-factors. We observed that both age and group were significantly associated with stage III gastric cancer survival (P < 0.05). Remarkably, the group had the highest statistical significance (*P* = 1.54E-14), suggesting that the stratification based on the features from all genes was an independent prognostic factor in stage III gastric cancer (**Table 2**).

**Table 2:** Multivariable Cox model with age, gender, race, and histology grade as co-factors.

Variables		Coef	exp(Coef)	se(Coef)	Z	P
Age		1.12E-04	1.00E+00	3.86E-05	2.896	0.00378
Gender	Male	4.12E-01	1.51E+00	2.69E-01	1.532	0.12547
Race	Black or African American	-1.13E-01	8.93E-01	7.00E-01	-0.162	0.87143
	White	4.04E-01	1.50E+00	3.83E-01	1.055	0.29138
Histology grade	G2	-1.82E-01	8.34E-01	1.05E+00	-0.173	0.86275
	G3	-8.96E-02	9.14E-01	1.03E+00	-0.087	0.93095
	GX	-1.54E+01	2.10E-07	3.40E+03	-0.005	0.99639
Group	Low-risk	-2.88E+00	5.59E-02	3.75E-01	-7.684	1.54E-14

### Prediction of the Underlying Mechanism of the lncRNAs in the Cox Model

As the lncRNAs could perform their function by interacting with proteins, we then predicted the interactions between the prognostic lncRNAs in the Cox model and proteins using a deep learning method, LncADeep. Moreover, we also conducted a correlation analysis between the proteins and lncRNAs. However, only one of the five lncRNAs in the Cox model, *CTD-2218G20.2*, was predicted to interact with 86 proteins (Pearson correlation coefficient, PCC > 0.3). The gene set enrichment analysis revealed that these interacting proteins also interacted with cancer-related proteins, including *KISS1, TIMP2, MMP11, IGFBP1, EGFR*, and *CDKN1C* (**Figure 4A**). Specifically, pregnancy-specific glycoproteins, including *PSG4, PSG5*, and *PSG7*, were those proteins jointly interacting with *CTD-2218G20.2* and cancer-related proteins, which were highly correlated with *CTD-2218G20.2* (**Figure 4B**, PCC > 0.3). These results suggested that *CTD-2218G20.2* might participate in the cancer progression *via* these cancer-related proteins.

## Discussion

Gastric cancer is a common malignant tumor with high occurrence and recurrence and is the leading cause of death worldwide (Bray et al., 2018). However, the prognostic value of protein-coding and non-coding RNAs in stage III gastric cancer has not been systematically analyzed. In this study, we identified 585 lncRNAs and 927 PCGs correlated with the overall survival rate of gastric cancer. Notably, 57.95% of PCGs and 68.72% of lncRNAs were positively correlated with the death rate in the Cox models (**Figure 1A**). To characterize the function of the prognostic genes, the prognostic genes positively or negatively correlated with death rates were subjected to KEGG enrichment analysis. Notably, the pathways of gap junction, focal adhesion, cell adhesion molecules (CAMs), and neuroactive ligand-receptor interaction were involved in the tumor microenvironments and cell-cell communications, suggesting that their dysregulation may promote the tumor progression. In accordance with previous studies (Wang et al., 2013; Yan et al., 2018; Zhao et al., 2019), the genes in gap junction, focal adhesion, and CAMs were significantly associated with gastric cancer prognosis. In addition, PI3K/Akt signaling pathway has been widely reported to regulate the tumorigenesis and progression (Tapia et al., 2014; Matsuoka and Yashiro, 2014) and act as a potential therapeutic target in gastric cancer (Ye et al., 2012; Singh et al., 2015).

To evaluate the performance of the prognostic genes in risk prediction, we built three Cox models based on prognostic lncRNAs, PCGs, and both (**Figure 3C**). The performances of the three models were systematically compared based on the criteria of log-rank test, Wald test, and C-index, which revealed that the features selected from all the prognostic genes showed higher performance than the features selected only from lncRNAs or PCGs. Furthermore, we investigated whether the stratification with the highest performance was a prognostic factor independent of clinical indicators, such as age, gender, race, and histology grade. The multivariable Cox regression analysis revealed that the stratification had the highest statistical significance (*P* = 1.54E-14), suggesting that the stratification based on the features from all genes was an independent prognostic factor in stage III gastric cancer. In addition, as the CEA and CA19-9 are commonly used biomarkers for gastric cancer risk prediction, we compared their prognostic values with those of the genes included in the multivariable Cox model. The hazard ratios (HR) of CEA and CA19-9 were estimated as 1.681 and 1.83 by meta-analysis (Song et al., 2015; Deng et al., 2015). However, the HR of CTD-2218G20.2 in the multivariable Cox model reached 3.48, suggesting that the lncRNA CTD-2218G20.2 was superior to the common clinical biomarkers like serum CEA and CA19-9.

Furthermore, we explored the underlying mechanism of the prognostic lncRNAs in the Cox model by predicting the lncRNA and protein interaction. Specifically, CTD-2218G20.2 was predicted to interact with 86 proteins (Pearson correlation coefficient, PCC > 0.3), some of which, including *PSG4, PSG5,* and *PSG7*, could also interact with cancer-related proteins, including *KISS1, TIMP2, MMP11, IGFBP1, EGFR*, and *CDKN1C* (**Figure 4A**). Notably, *KISS1, TIMP2, MMP11, IGFBP1,* and *EGFR* have been reported to be involved in the metastasis of gastric cancer (Guan-Zhen et al., 2007; Kou et al., 2013; Wang et al., 2017; Wang et al., 2018; Sato et al., 2019). These results suggested that *CTD-2218G20.2* might participate in the cancer progression *via* these cancer-related proteins.

The present study still had some limitations, such as lack of experimental validation or large sample size. However, we aimed to discover some key prognostic PCGs and lncRNAs in stage III gastric cancer that could not be extrapolated to early stage GC patients. In summary, this systematic analysis of the prognostic lncRNAs and PCGs was of great importance to the understanding of the progression of stage III gastric cancer.

## Data Availability Statement

All datasets generated and analyzed for this study are included in the article/**Supplementary Material**.

## Ethics Statement

No identifiable data is present in this paper. 

## Author Contributions

Conception and design: XS. Administrative support: XS. Provision of study materials or patients: XS, JZ, and WY. Collection and assembly of data: all authors. Data analysis and interpretation: all authors. Manuscript writing: all authors. Final approval of manuscript: all authors.

## Funding

This work was funded by a Key Project of Natural Science Foundation of Liaoning Province (NO. 20170540566).

## Conflict of Interest

The authors declare that the research was conducted in the absence of any commercial or financial relationships that could be construed as a potential conflict of interest.
